# Acute Renal Failure Secondary to Tuberculosis: A Diagnostic Challenge

**DOI:** 10.1155/2012/510179

**Published:** 2012-02-22

**Authors:** Saeed I. Khilji, Hong Kuan Kok, Limy Wong, Anthony M. Dorman, J. Joseph Walshe

**Affiliations:** ^1^Department of Nephrology, Beaumont Hospital, Dublin 9, Ireland; ^2^Department of Renal Pathology, Beaumont Hospital, Dublin 9, Ireland

## Abstract

Tuberculosis is a multiorgan disease with varied clinical presentations and is reemerging due to increasing immigration and globalization. We present the case of an immigrant female patient who developed acute renal failure with clinical and biochemical features suggestive of lupus nephritis but with a timely renal biopsy showing caseating granulomata in the renal parenchyma consistent with renal tuberculosis. Despite treatment with antituberculosis treatment and resolution of TB on repeat renal biopsy, she remained haemodialysis dependent. We discuss the diagnostic challenges faced in this presentation and also explore possible differential diagnoses. This rare presentation highlights the importance of renal biopsy in the diagnosis and treatment of acute renal failure and the atypical presentation of tuberculosis.

## 1. Introduction

Tuberculosis (TB) is often seen as a disease of the past but has been reemerging in recent times [1]. The contemporary disease patterns and clinical presentations may be altered due to factors such as acquired immunodeficiency syndrome (AIDS) and globalization with immigration from TB endemic countries in Eastern Europe, Asia, and Africa. We report on an unusual case of acute renal failure secondary to renal tuberculosis where the initial clinical and serological picture was highly suggestive of lupus nephritis. We also briefly discuss the challenges in the diagnosis and management of this clinical presentation.

## 2. Case Presentation

A 45-year-old female resident in Ireland, originally from the Philippines, was transferred to our institution for further management of acute renal failure. She initially presented to the referring hospital with generalized malaise and fatigue over the preceding month associated with a small, painless swelling on the left side of the neck. She denied any fevers, chills, rigors, night sweats, photosensitive rash, joint pains, weight loss, or urinary tract symptoms. She did not suffer from any upper respiratory tract symptoms, sinusitis, or rash. She was not taking any medications or herbal remedies. Her past medical history was unremarkable except for lumpectomy for a benign breast cyst several years ago. Her menstrual cycles were normal and there was no history of miscarriages in the past. Her serum creatinine on admission was 205 *μ*mol/L with an active urine sediment.

On examination, she appeared unwell, was afebrile, with a blood pressure 130/80 mmHg, and weighed 57 kgs. A bedside urine dipstick was positive for blood (+) and protein (+++). There was no evidence of oral ulceration, rashes, or joint swelling. Her neck was supple to palpation except for a solitary 1 cm nontender, mobile lymph node in the left anterior triangle of neck. The thyroid gland was not enlarged. The remainder of her cardiovascular, respiratory, and abdominal examination was unremarkable.

Biochemical investigations revealed a serum creatinine which had risen to 568 *μ*mol/L and urea of 23.4 mmol/L. Haematological parameters showed a normal haemoglobin level of 13 g/dL with a serum C-reactive protein of 74 mg/L (normal < 10). No prior biochemical parameters were available for comparison. A full vasculitic screen was performed; antinuclear factor (ANF) was positive with a homogenous pattern, antidouble-stranded DNA antibodies were raised (anti-dsDNA titre 59, >30 positive) and antineutrophil cytoplasmic (ANCA) and antiglomerular basement membrane antibodies were negative. Antistreptolysin O titres were also within normal limits. Serum complement levels showed depletion of C4 and normal C3 levels. Serology was negative for human immunodeficiency virus (HIV), toxoplasmosis, and brucellosis. A 24-hour urine collection quantified the proteinuria at 1.2 g/24 hrs.

**Figure 1:**
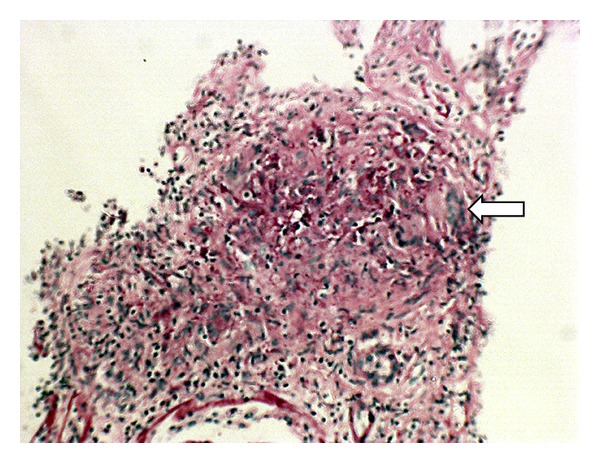
High-power haematoxylin- and eosin-stained renal biopsy before antituberculous treatment demonstrates the presence of a granuloma with caseating central necrosis (white arrow).

Her admission chest radiograph was normal. Renal ultrasonography revealed normal sized kidneys with good corticomedullary differentiation and no evidence of hydronephrosis. Fine needle aspiration biopsy of the neck lump showed mature lymphocytes admixed with neutrophils, with 90% of the cells showing positivity for CD45—strongly suggestive of nonspecific inflammatory changes. In view of the presentation of acute renal failure, haematuria, and proteinuria with serological evidence suggestive of lupus, a renal biopsy was performed to clarify the diagnosis. Histopathology results, however, showed widespread caseating granulomata with Langerhan's cells (Figure 1) consistent with renal tuberculosis. No acid fast bacilli (AFB) were seen on light microscopy.

On further review, our patient had been vaccinated with BCG in her childhood. She could not recall any contact with tuberculosis carriers nor was there any history of recent travel abroad. A mantoux test was subsequently performed and was positive at 12 mm. Sputum microscopy was negative for AFB. Serial early morning urine samples were also examined and cultured for the presence of AFB but were negative. In view of the renal biopsy findings, a CT of the thorax, abdomen, and pelvis was performed which revealed enlarged retrogastric lymph nodes measuring up to 2.8 × 1.2 cm and further subcentimetre mediastinal and retroperitoneal lymph nodes.

**Figure 2:**
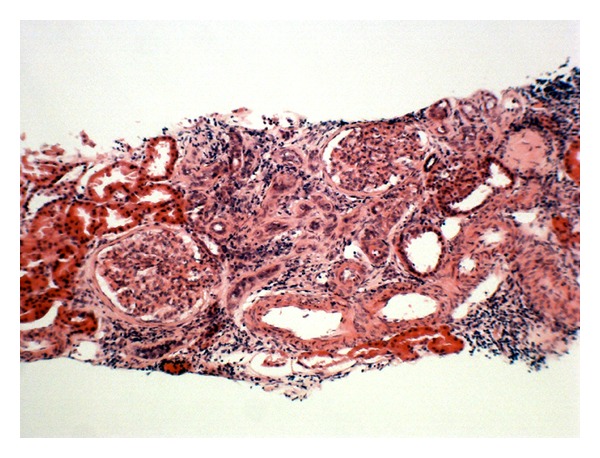
High-power haematoxylin- and eosin-stained renal biopsy after completion of antituberculous treatment shows tubulointerstitial fibrosis in the absence of caseating granulomas.

She was commenced on standard combination antituberculous chemotherapy consisting of isoniazid, rifampicin, ethambutol, and pyrazinamide. She subsequently developed deranged liver enzymes which normalized following withdrawal of rifampicin. Our patient completed 18 months of treatment for renal tuberculosis, but failed to recover renal function and is now dialysis dependent, awaiting renal transplantation. A repeat renal biopsy was performed following completion of anti-tuberculous therapy. This confirmed significant tubulointerstitial fibrosis in the absence of any caseating granulomas (Figure 2). A follow-up CT scan showed resolution of the previously detected lymphadenopathy.

## 3. Discussion

The case above illustrates the presentation of acute renal failure in a patient with an active urinary sediment and serological evidence of lupus as suggested by anti-dsDNA positivity and complement consumption. These biochemical features strongly raised the clinical suspicion of lupus nephritis. However, other causes of rapidly progressive renal failure including postinfectious glomerulonephritis as well as vasculitic and interstitial glomerulonephritis were also considered in the differential diagnosis. The renal impairment in this case was felt to be acute rather than acute on chronic kidney disease in view of the rapid deterioration in her renal function, absence of any history of prior renal disease, normal renal size and echogenicity at ultrasonography and absence of anaemia at presentation. Our patient also presented with painless cervical lymphadenopathy, and other conditions such as lymphoma, sarcoidosis, toxoplasmosis, brucellosis, HIV, local salivary gland neoplasia and paragangliomas were also considered. However, a renal biopsy was critical in securing the correct diagnosis of renal tuberculosis to guide appropriate treatment in our patient. The presence of caseous necrosis on renal biopsy made sarcoidosis unlikely in this setting.

This case highlights the importance of postprimary tuberculosis as a reemerging clinical problem, particularly with the trend of widespread immigration and globalisation [2]. Renal tuberculosis usually presents as gross haematuria, insidious pyuria, or obstructive uropathy but can also present rarely with acute renal failure in HIV patients with tuberculosis treated with rifampicin [3–5]. To our knowledge, only one case of tuberculous granulomatous interstitial nephritis causing acute renal failure and proteinuria has been reported previously [6]. This case is unique in that it combined a compatible clinical and serological picture suggestive of lupus nephritis, while renal histopathology confirmed the cause of acute renal failure as tuberculosis. Although highly specific for systemic lupus erythematosus, false positive anti-dsDNA antibodies have been described in a healthy subset of an elderly population but are very unusual in younger individuals such as our patient. The mechanism of this finding remains unclear [7]. The degree of proteinuria in our case is also unusual in renal tuberculosis, however, there were no other clinical symptoms or signs to support lupus at diagnosis and these did not manifest in her later followup as well.

To our knowledge, acute renal failure secondary to renal tuberculosis presenting in this manner has not been reported previously in the literature. This case highlights the importance of clarifying the diagnosis of acute renal failure through renal biopsy where possible, prior to the commencement of treatment. It also illustrates the need to be aware of atypical clinical presentations of tuberculosis which may be more common in the contemporary setting.

## 4. Conclusion

The diagnosis of tuberculosis and systemic lupus erythematosus can be clinically challenging as highlighted by this case. It is also important to consider tuberculosis in the differential diagnosis of unusual presentations, particularly in immigrant populations and immunosuppressed individuals.

## References

[1] Sohail M (2006). Tuberculosis: a re-emerging enemy. *Journal of Molecular and Genetic Medicine*.

[2] Porter JDH, McAdam KPWJ (1994). The re-emergence of tuberculosis. *Annual Review of Public Health*.

[3] Costiniuk CT, Mccarthy AE, Talreja H (2011). Acute renal failure and disseminated intravascular coagulation associated with rifampin in tuberculosis treatment. *International Journal of Tuberculosis and Lung Disease*.

[4] Fallouh B, Nair H, Langman G, Baharani J (2010). The Case Unusual cause of acute renal failure in a patient with HIV. *Kidney International*.

[5] Simon HB, Weinstein AJ, Pasternak MS (1977). Genitourinary tuberculosis. Clinical features in a general hospital population. *American Journal of Medicine*.

[6] Sampathkumar K, Sooraj YS, Mahaldar AR (2009). Granulomatous interstitial nephritis due to tuberculosis-a rare presentation. *Saudi Journal of Kidney Diseases and Transplantation*.

[7] Ruffatti A, Calligaro A, Del Ross T (1990). Anti-double-stranded DNA antibodies in the healthy elderly: prevalence and characteristics. *Journal of Clinical Immunology*.

